# An Assessment of Real-World Evidence and Other Sources Supporting Payer Coverage Decisions for Pharmacogenomic Testing in Psychiatry

**DOI:** 10.3390/jpm15060232

**Published:** 2025-06-03

**Authors:** Sandra E. Yankah, Maryam Nafie, Rachele M. Hendricks-Sturrup, Christine Y. Lu

**Affiliations:** 1 Duke-Robert J. Margolis, MD, Institute for Health Policy, Washington, DC 20004, USA; seyankah1@gmail.com (S.E.Y.); maryam.nafie@duke.edu (M.N.); 2 School of Pharmacy, Faculty of Medicine and Health, The University of Sydney, Sydney, NSW 2006, Australia; christine.lu@sydney.edu.au; 3 Kolling Institute, The University of Sydney Faculty of Medicine and Health and the Northern Sydney Local Health District, Sydney, NSW 2065, Australia; 4 Harvard Medical School, Boston, MA 02115, USA; 5 Department of Population Medicine, Harvard Pilgrim Health Care Institute, Boston, MA 02215, USA

**Keywords:** pharmacogenomics, psychiatry, payer coverage, precision medicine, real-world evidence

## Abstract

**Background:** Sources and evidence cited to inform payer coverage decisions on pharmacogenomic (PGx) testing in psychiatry are presently underexplored. **Methods:** We conducted a qualitative and quantitative assessment of publicly available coverage policies from 14 US payers, examining the number and both the type and source of citations across policies and coverage decisions. Payers were classified as for-profit or mutual fund versus non-profit or government, and their coverage decisions were categorized as either coverage (limited or specified) or no coverage. **Results:** Among 32 unique sources cited, peer-reviewed literature as a single source was most frequently cited across all policies. Of 207 peer-reviewed papers cited across all policies, 40% (*n* = 83) were psychiatry-specific real-world evidence (RWE) studies. No statistically significant relationships were observed when comparing variance in the number of citations per policy by payer type (*p* = 0.22) or coverage decision (*p* = 0.75; unadjusted variance of 61.25 and an adjusted variance of 60.98 for both comparisons). For-profit or mutual fund payers and/or payers providing no coverage cited systematic reviews and non-randomized controlled cohort RWE studies most often. Non-profit or government payers and/or payers providing coverage cited case series or case-control RWE studies most often. Six psychiatry-specific RWE studies and contributions from 13 distinct sources were often cited, regardless of payer type or coverage decision. **Conclusions:** RWE, among several sources, are cited in many forms and to varying degrees among payers providing coverage decisions for PGx testing in psychiatry, with coverage determinations being largely based on how certain payers interpret evidence on the clinical value of testing.

## 1. Introduction

Pharmacogenomic (PGx) testing is a molecular diagnostic strategy that tailors medication and dosage selection based on a patient’s genetic profile or drug metabolism phenotype. Accumulating research indicates that PGx testing in psychiatric care settings has the potential to improve the pharmacological management of mental health conditions by optimizing medication and dosage selection, decreasing adverse drug events, and reducing healthcare spending per patient [1]. In fact, the Clinical Pharmacogenetics Implementation Consortium (CPIC) offers prescribing guidelines based on substantial high to moderate evidence for seven (7) psychiatric drugs with US Food and Drug Administration (FDA) labeling (i.e., CPIC Level A status for PGx biomarkers *CYP2D6* and *CYP2C19* and respective drug-gene pairs with citalopram, escitalopram, amitriptyline, atomoxetine, nortriptyline, paroxetine, and vortioxetine; see Supplementary File S1 in Supplementary Materials).

In additional to variable clinical recommendations from the above sources, access to and adoption of PGx testing in psychiatric care settings vary significantly due to a myriad of patient, provider, and health system factors, with cost being a major contributor [2]. Moreover, recent conversations among payers, bioethicists, and clinicians reflect ongoing uncertainty about the value and clinical utility of PGx testing in psychiatry [3]. Current psychiatric treatment often relies on both trial and error and polypharmacy approaches, further complicating efforts to establish PGx testing as standard care. While further evidence is needed to validate the real-world effectiveness of PGx testing psychiatry, the pace of evidence generation is closely tied to implementation factors such as insurance coverage. For many patients, access to genetic testing is primarily determined by cost, making insurance coverage a critical factor in ensuring the affordability of clinical-grade testing.

A prior analysis found variations in insurance coverage for PGx testing in oncology, with the evidence cited within insurance coverage policies playing a significant role in influencing payer coverage decisions [4]. In oncology, payer decisions often reflect the availability of strong clinical evidence—especially randomized controlled trials and large-scale real-world studies—that demonstrate consistent benefit and are backed by clinical guidelines or regulatory endorsements. These features help create a clearer path to coverage. In contrast, evidence supporting PGx testing in psychiatry is more diffuse if not inconclusive as evidence bases supporting the utility of PGx testing in psychiatry remain provisional or under development for 32 drug–gene pairs (see Supplementary File S1 in Supplementary Materials).

Considering this, there is a present lack of research directly examining payer coverage decisions for PGx testing in psychiatry and sources and evidence cited in those decisions. To address this gap, we evaluated citations listed within and across a national sample of payer coverage policies and characterized evidence cited and organized by US payer types and decisions to better understand the role of evidence and types of evidence in coverage decisions for testing in psychiatry [5,6]. We did not assess the rationale behind payer evidence selection and underlying decisions in each payer policy. Specifically, we assessed payer types and coverage decisions for PGx testing in psychiatry, categorized evidence, and sources cited in policies to support payers’ coverage decisions, assessed the potential relationships that exist between payer type or coverage decisions and the number of sourced cited, and, lastly, summarized evidence cited in coverage policies.

## 2. Methods

### 2.1. Payer Policy Search and Selection

A systematic search was conducted in October 2024 online via Google, payer websites, and a commercial database (PolicyReporter, Morrisville, NC, USA) using any combination of the following terms to identify US payer coverage policies effective during that time: pharmacogenomic, pharmacogenetic, genetic, psychiatry, mental, behavioral, coverage, policy, and insurance. Information sought/sourced publicly available payer coverage policies from national, regional, state, and local payers in the US.

Our search initially identified 14 unique payers and 38 payer plans serving Medicare, Medicaid, individual, commercial (parent company and subsidiaries), self-funded or employer-sponsored, or unspecified markets. From there, policies were consolidated to ensure adequate representation across national, regional, state, and institutional payers, market share, and for-profit versus non-profit status. Policies were included in the sample if the policy represented the parent company (commercial payers only) and contained a policy effective date, a PGx test, or Current Procedural Terminology code (billing code for medical services and procedures) relevant to psychiatry or a biomarker with a current PGx US Food and Drug Administration labeling section [7], and a coverage decision for a single or multi-panel genetic test with claims of effectively guiding psychiatric medication selection or dosage was specified in the policies.

Policies focused specifically on genetic testing for intellectual disabilities (e.g., autism spectrum disorder), substance use, or abuse disorders (e.g., prediction for opioid use disorder), or central nervous system disorders associated with age-related decline (e.g., dementia) were excluded from the analysis.

### 2.2. Policy Content Analysis

The following information was extracted and catalogued from each policy selected for inclusion to closely evaluate evidence base and sources cited alongside coverage decisions:

▪ Policy type (general or psychiatry specific)
▪ Payer type
  - For-profit or mutual fund: entity with a tax filing status based on individual shareholder investments or an investment fund.
  - Non-profit or government: entity with a tax filing status based on public benefit, charity, or social cause.
▪ Coverage determination
  - No coverage
  - Coverage
    ○ Specified (i.e., coverage for a specific test/subpopulation).
    ○ Conditional (i.e., coverage based on meeting clinical or prior authorization criteria).
▪ Active company subsidiaries (when applicable and when information was freely available online) to exclude non-parent or subsidiary policies with redundant or boilerplate language seen in parent company policies (commercial payers only).
▪ References cited
  - For policies specific to PGx testing for mental health or psychiatric purposes, all references were quantified and catalogued.
  - For policies non-specific to PGx testing for mental health or psychiatric purposes, only references relevant to mental health or psychiatry were quantified and catalogued.

### 2.3. Evaluation of Payer Types and Coverage Decisions

Payers were categorized by type (for-profit or mutual fund versus non-profit or government) and analyzed coverage decisions (covered [conditional or specified] versus not covered). Summary statistics (the average number of citations) were obtained for general comparison (for-profit or mutual fund versus non-profit or government; covered [conditional or specified] versus not covered) using Microsoft Excel. Given, the non-normal distribution of data, Wilcoxon rank-sum (Mann–Whitney) tests were conducted at a 95% confidence interval (CI) using STATA to determine if statistically significant relationships exist between payer type/coverage decision and the number of sources cited.

### 2.4. Assessment of Sources Cited in Payer Policies

Peer-reviewed sources cited in payer policies were further evaluated by two authors (R.M.H.-S. and M.N.) along the following criteria until >95% agreement was reached, with a third author (C.Y.L.) available to resolve potential disagreement and assess inter-rater reliability: publication year, Oxford Centre for Evidence-Based Medicine 2011 (OCEBM) Evidence Level (see Supplementary File S2 in Supplementary Materials), psychiatry-specific, and classification as an RWE study according to a detailed definition of RWE and RWE classification criteria described by Rahman et al. [8,9]. There is increased interest and consideration among regulators, payers, and health systems in using RWE to address research questions concerning the clinical and economic utility of medical products, including but not limited to PGx testing [9,10,11,12]. For this reason, a descriptive summary of peer-reviewed sources classified as RWE studies and psychiatry-specific studies was generated to determine OCEBM Level of Evidence therein and date range. Microsoft Excel software was used to assess and summarize quantitative findings and key findings from the most commonly cited psychiatry-specific RWE studies.

### 2.5. Ethics Statement

Our work was not intended as human subjects research and solely involved a review of publicly available information online. Payer entity names were omitted for privacy and can be made available upon direct request.

## 3. Results

### 3.1. Payer Assessment and Coverage Analysis

Upon closely evaluating payer policies to exclude non-parent/subsidiary policies with redundant or boilerplate language seen in parent company policies (commercial payers only), our final analyses included 14 unique payers and policies. Variations in coverage for PGx testing were observed (no coverage [*n* = 7], conditional coverage [*n* = 3], and specified coverage [4]) among for-profit or mutual fund (*n* = 7) and non-profit or government (*n* = 7) payers. These policies together had 346 total citations from 32 distinct sources across all payer policies applicable to PGx testing in psychiatry (see Table 1). The number of citations per unique payer ranged from zero (0) to 77.

### 3.2. Statistical Assessment of Sources Cited

Statistical analyses showed high variation in the mean number of references cited according to payer type and coverage decision, as well as comparatively large and unequal variance in number of sources cited across policies, but these were not statistically significant. An average of 30.71 references were cited by for-profit or mutual fund compared to an average of 18.71 references cited by non-profit or government payers. An average of 28.86 references were cited by payers providing some form of coverage compared to 28.57 references cited by payers providing no coverage.

On average, 24.71 citations were observed across all policies (95% CI: 10.9 to 38.53) with variance in the number of references cited per policy and across all policies, regardless of payer type and coverage decision, being 572.37 (95% CI: 300.82 to 1485.57). When comparing the number of citations per policy according to payer type, Mann–Whitney tests showed a non-statistically significant (*p* = 0.22) unadjusted variance of 61.25 and an adjusted variance of 60.98. Upon comparing the number of citations per policy according to coverage decision, Mann–Whitney tests showed a higher level of statistical insignificance (*p* = 0.75) for the same unadjusted variance of 61.25 and an adjusted variance of 60.98.

### 3.3. Assessment of Sources Cited Across Payer Types

We observed overlap in terms of for-profit or mutual fund and non-profit or government payers citing at least one (1) reference from the same source. Specifically, both for-profit or mutual fund and non-profit or government payers cited work from the following eleven (11) sources: US Centers for Medicare and Medicaid Services (CMS), US Centers for Disease Control and Prevention (CDC), US FDA, UptoDate, PharmGKB, industrial or market solutions (which do not disclose or cite discernable evidence), Clinical Pharmacogenetics Implementation Consortium (CPIC), Canada’s Drug Agency (CDA) formerly known as Canadian Agency for Drugs and Technologies in Health (CADTH), Association for Molecular Pathology and/or College of American Pathologists (AMP/CAP), payer health guidelines, and subject matter panel and advisory committees (see Table 1). Payers providing some form of coverage or no coverage cited work from the following thirteen (13) sources: US CMS, US CDC, US FDA, UptoDate, National Institute of Health (NIH), PharmGKB, Industrial or market solution, CPIC, CADTH, AMP/CAP, American College of Medical Genetics and Genomics (ACMG), payer health guidelines, and subject matter panel and advisory committees (see Table 1).

### 3.4. Assessment of Peer-Reviewed Literature Cited Across Payer Types

Peer-reviewed literature was cited most frequently, compared to all other reference sources, and in similar proportion among both for-profit or mutual fund and non-profit or government payer types (132 out of 215 total references [61%] and 75 out of 131 total references [57%], respectively). Likewise, peer-reviewed literature was most cited across all payers, with payers providing no coverage cited peer-reviewed literature at a higher proportion (129 of 200 [65%]) than payers providing some form of coverage (78 of 146 [53%]).

### 3.5. Assessment of RWE in Peer-Reviewed Literature Cited Across Payer Types

**Figure 1:**
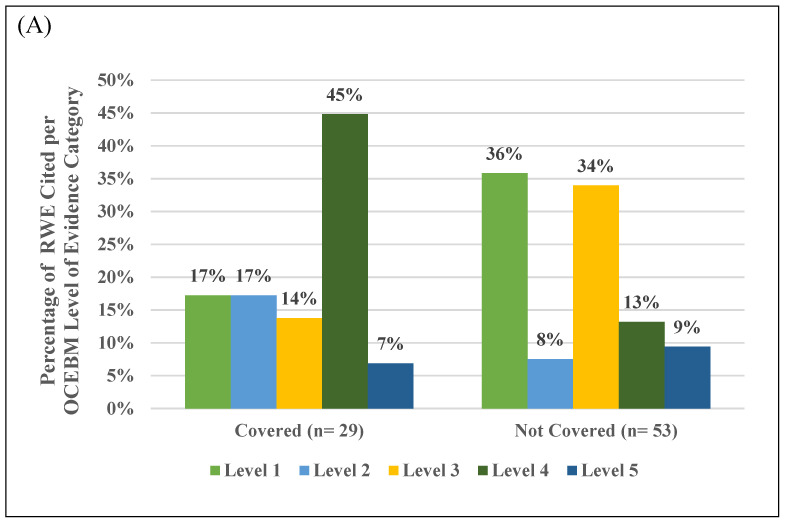

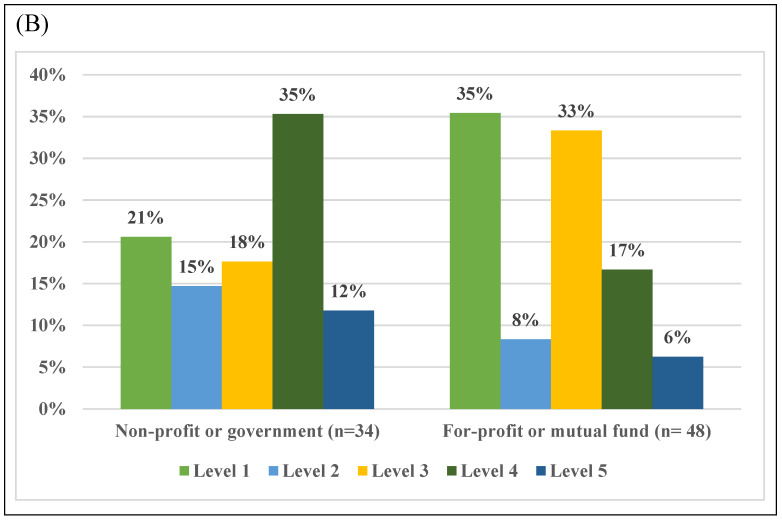
Summary of real-world evidence (RWE) cited in payer policies for PGx testing in psychiatry, categorized by Oxford Centre for Evidence-Based Medicine 2011 (OCEBM) Level of Evidence category and stratified by coverage decision (**A**) and payer type (**B**). Level 1: Systematic review of studies reflecting local and current random sample or censuses. Level 2: Systematic review of surveys that can be matched to local circumstances (includes randomized trials and observational studies with dramatic effects). Level 3: Local non-random sample studies (includes cohort studies and non-randomized controlled cohorts). Level 4: Case series studies (includes case-control and historically controlled studies). Level 5: Studies reflecting mechanism-based reasoning.

Of all peer-reviewed article citations (*n* = 207), 82 citations were determined upon assessment by the authors (R.M.H.-S. and M.N.) as psychiatry-specific RWE studies (40%). Publication years for these studies ranged from 2005 to 2024 and publications spanned OCEBM Levels of Evidence ranging from 1 to 5 (see Figure 1). When stratified for coverage decision (Figure 1A), payers choosing to cover testing most frequently cited RWE studies at OCEBM Level of Evidence 4 (13 out of 29 citations). Payers declining coverage most frequently cited RWE studies at OCEBM Levels of Evidence 1 and 3 (19 out of 53 and 18 out of 53 citations, respectively). When stratified by payer type (Figure 1B), non-profit or government payers most frequently cited RWE studies at OCEBM Level of Evidence 4 (12 out of 34 citations). For-profit or mutual fund payers most frequently cited RWE studies at OCEBM Levels of Evidence 1 and 3 (17 out of 48 and 16 out of 48 citations, respectively).

We identified six (6) psychiatry-specific RWE studies cited more than once and in more than one payer policy and within the most frequently categorized OCEBM levels in Figure 1 (i.e., studies categorized in Levels 1, 3, and 4; PMID 22198443, 23047243, 24018772, 25686762, 26445691, and 29690793; see Table 2) [13,14,15,16,17]. Key findings from each study identified are summarized in Table 2. No studies were identified based on this description at OCEBM Level 4. With the exception of one (1) paper (PMID 22198443, OCEBM Evidence Level 3, Not Covered), most papers (*n* = 5) cited were accompanied by either a Covered or Not Covered payer decision and published between 2021 and 2018 [13]. Also, with the exception of one (1) paper (PMID 29690793, OCEBM Evidence Level 1, Both Covered and Not Covered), most papers (*n* = 5) conveyed results indicating therapeutic benefit associated with PGx testing (i.e., changes in medication dose due to PGx biomarker status, reduction in psychiatric symptoms and medication side effects following PGx-guided treatment, measurable effect of PGx-guided treatment based on treatment severity, and increase in quality of life [12].

## 4. Discussion

Here, we report high variance in the number of citations per payer coverage policy for PGx testing, regardless of payer type and coverage decision. Although no statistically significant relationships were observed between payer type or coverage decision and the number of references cited per payer policy, a higher level of statistical insignificance was observed when assessing this relationship according to coverage decision (versus payer type). We also observed that payers may consider a variety factors, including but not limited to peer-reviewed studies containing RWE, to support their decisions around coverage for PGx testing in psychiatry. Next, we observed that non-profit or government payers often cited RWE studies at OCEBM Level of Evidence 4, whereas for-profit or mutual fund payers often cited RWE studies at OCEBM Levels of Evidence 1 and 3. Payers choosing to cover testing often cited RWE studies at OCEBM Level of Evidence 4, whereas payers declining coverage often cited RWE studies at OCEBM Levels of Evidence 1 and 3. Lastly, where matters of payer alignment might arise, both for-profit or mutual fund and non-profit or government payers cited references from the same 11 same sources (US CMS, US CDC, US FDA, UptoDate, PharmGKB, industrial or market solutions, CPIC, CDA, AMP/CAP, payer health guidelines, and subject matter panel and advisory committees). Payers, regardless of type or coverage decision, cited six (6) psychiatry-specific RWE studies more than once (PMIDs 22198443, 23047243, 24018772, 25686762, 26445691, and 29690793).

Alongside these findings, we provide the scientific community with a methodological approach that may be useful to help individuals understand evidence and other sources cited in payer coverage policies for PGx testing in psychiatry. We anticipate that these methods can be applied across other therapeutic areas outside of psychiatry and for similar investigational purposes, particularly in situations where patients are unable to obtain direct to consumer (DTC) or cash price negotiation pathways to access PGx testing.

Notwithstanding one payer with zero sources cited and regardless of payer type, payers cited peer-reviewed literature most frequently in their policies, demonstrating general payer alignment around the use of peer-reviewed literature to substantiate coverage decisions. We observed that for-profit or mutual fund payers or payers providing no coverage for PGx testing cited peer-reviewed literature most often and psychiatry-specific RWE studies at OCEBM Levels of Evidence 1 and 3 more frequently than payers providing coverage. Non-profit or government payers or payers providing coverage for PGx testing most frequently cited psychiatry-specific RWE studies at OCEBM Levels of Evidence 4. These patterns suggest that for-profit or mutual fund payers and payers denying coverage may either weigh or favor interpretations based on a totality of evidence (i.e., systematic reviews) and RWE generated through non-randomized controlled cohort studies more than non-profit or government payers and payers providing coverage who may either weigh or favor RWE in case series studies.

Regardless of payer type or coverage decision, payers seemed to consult many of the same additional sources (i.e., US CMS, US CDC, US FDA, UptoDate, PharmGKB, etc.). Also, among the six studies cited at the most common Oxford Centre for Evidence-Based Medicine 2011 (OCEBM) Evidence Level, classified as both RWE studies and psychiatry-specific, and with two or more citations among payers in our sample, no specific patterns were observed with respect to a payer coverage decision based on key study outcomes or conclusions. Yet, given that payers deciding to cover or not cover testing frequently cited these six RWE studies, it is possible that these studies might serve as useful sources to support engagement among payers. This could be useful to support payer engagement as (1) payers continue to discuss or deliberate evidentiary needs and considerations for PGx testing in psychiatry, and (2) as evidence on the clinical utility of PGx testing in psychiatry continues to develop.

Overall, our findings underscore the need for solutions that have been proposed in prior work, such as the development of standardized data and evidence review processes among payers, payer engagement in RWE study design, use of incentives and partnerships to lower barriers to RWE generation, education of payers and providers concerning the use of RWE and PGx testing, learning payer preferences for RWE with respect to PGx testing, and frameworks for conducting outcome-based contracting for PGx testing [12,18,19]. In addition, given that clinical outcomes appear to be a key consideration for payers, future RWE studies should prioritize investigating the impact of PGx testing on psychiatric treatment selection and outcomes, particularly for specific beneficiary populations rather than the total population [18,20,21].

As efforts grow to engage payers more directly in the design and execution of real-world evidence (RWE) studies, it is important to remain attentive to the potential for bias that such collaborations might introduce. Payers can bring practical insight into coverage criteria and patient access, which can potentially strengthen the relevance of study design. However, their involvement could also lead to concerns about undue influence on study outcomes or selective emphasis on evidence that aligns with business objectives. To mitigate these risks, future research partnerships should prioritize independent oversight, transparency in protocol development, and pre-specified analytic plans. Multi-stakeholder governance models that include neutral third parties—like academic researchers, clinicians, and patient representatives—may help ensure that payer priorities inform but do not dominate the scientific process. Establishing clear boundaries around payer involvement can foster mutual trust while safeguarding the integrity and credibility of the resulting evidence base.

Amid growing demand for public transparency, and as beneficiaries may have questions or hold concerns about how payer entities engage in the selection, listing, or preponderance of scientific evidence, the presence or absence of certain economic or business interests that could favor a specific payment approach holds tremendous beneficiary and provider interest. Payers, like clinicians, hold an obligation or right to deny any rendering of services, including molecular testing, where risks of potential harm due to conflicting or inconclusive evidence, and thus a lack of interpretation around medical necessity, outweigh the uncertainty of benefit. Patients today, however, have access to DTC genetic testing mechanisms that allow market demand and forces to elevate the patient, if not solely clinical, value of PGx testing in psychiatry. Amid this push-and-pull dynamic between payers and patients, prescribing clinicians have an ethical obligation to withhold recommendations for testing that could pose unnecessary or disproportionate risks of harm (e.g., health risk due to prescribing or de-prescribing) while also respecting and upholding competent patient autonomy. All these factors should be considered to mitigate or avoid any negative influence on the scientific integrity of future investigations that will involve payer engagement in RWE development and implementation and that affect clinical care or patient choices.

Today, and as observed herein, payers consider systematic reviews and other study types, all of which can be evaluated or disseminated within emerging or existing evidentiary frameworks that serve to enhance study rigor and reproducibility and control for bias. Frameworks include, for example, the Institute for Clinical and Economic Review’s evidence rating matrix, which exists alongside its industry partnership to generate “decision-grade” RWE and mission to “expand use of RWE to complement other sources of information used in its value assessments” [22,23]. Also, an RWE study registry exists under our Real-World Evidence Transparency Initiative, whereas all study registrations therein require an uploaded study protocol, and registrants are encouraged to follow a study template developed by the International Society for Pharmacoepidemiology (ISPE) and the Professional Society for Health Economics and Outcomes Research (ISPOR) joint task force that has been endorsed and recently adapted by the US CMS for public consideration and comment [21,24]. For this reason, payers might consider evidence evaluated under these rigorous frameworks and registries that may endorse them [25].

Moving forward, it will be important to consider our findings herein to support this ongoing work and related initiatives focused on building a general understanding of whether or how, and the process through which, payers engage in the consideration and subsequent selection or development of (real-world) evidence. Clinical practice areas where PGx testing is relevant, such as psychiatry, would be a key clinical and therapeutic use case for consideration, given its complexity, for payers managing pharmacy care [20]. Importantly, future work should also explore payer rationale for our observed administrative and stylistic differences in payer policy drafting and online publication of evidentiary sources on PGx testing in psychiatry.

Our findings are accompanied by limitations that should be noted to inform both practice and future work. First, payers may require additional payment guidelines or prior authorizations for coverage that are not captured in this analysis. Also, parent companies may have subsidiary plans that might also vary in terms of coverage and sources cited in their policies, especially in cases where subsidiary plans are population-specific. Thirdly, our findings are exclusive to the practice of psychiatry and may, therefore, not be generalizable to other clinical or therapeutic areas outside of psychiatry. Lastly, coverage is often mandated based on applicable legal requirements of a state or the federal government, which might also affect local coverage determinations; this analysis does not capture these. We note that for certain areas outside the immediate scope of our present analysis (i.e., rationale behind payer evidence selection and decisions and assessment of methodological rigor and quality of each article included in each payer policy), future qualitative or contextualizing work is needed.

## 5. Conclusions

Our findings highlight substantial variation among payers in both coverage decisions and sources and evidence cited concerning the clinical utility of PGx testing in psychiatry. Peer-reviewed studies of various OCEBM Evidence Levels, inclusive of RWE, are highly cited among payers to substantiate their coverage decisions for PGx testing psychiatry. The observed variation in payer coverage and underlying rationale for coverage can be expected at this time given that relevant, reliable, and conclusive RWE on PGx testing in psychiatry is still emerging. Our assessment supports broader efforts and lays foundational work toward an understanding of payer perspectives and preferences for key sources and evidence concerning the clinical value of PGx testing in psychiatry.

## Figures and Tables

**Table 1:** Overview of number and source of citations across payer coverage policies for pharmacogenomic (PGx) testing in psychiatry, organized by payer type and by coverage decisions.

	Number of References per Payer Type (For-Profit or Mutual Fund [n = 7] and Non-Profit or Government [n = 7]) and per Coverage Decision (Covered [n = 7] and no Coverage [n = 7])			
Source Cited (Total of 32)	For-Profit or Mutual Fund (215 Total References)	Non-Profit or Government (131 Total References)	Covered (Specified or Partial; 146 Total References)	Not Covered (200 Total References)
Centers for Medicare and Medicaid Services (CMS; 16 total citations)	4 (2%)	12 (9%)	11 (8%)	5 (3%)
Centers for Disease Control and Prevention (CDC; 8 total citations)	3 (1%)	5 (4%)	6 (4%)	2 (1%)
United States (US) Food and Drug Administration (FDA; 14 total citations)	10 (5%)	4 (3%)	5 (3%)	9 (5%)
UptoDate (4 total citations)	3 (1%)	1 (1%)	1 (1%)	3 (2%)
National Institute of Health (NIH; 9 total citations)	9 (4%)	0 (0%)	3 (2%)	6 (3%)
Department of Energy (2 total citations)	0 (0%)	1 (1%)	1 (1%)	0 (0%)
Federal Register (1 total citation)	1 (0.5%)	0 (0%)	0 (0%)	1 (0.5%)
PharmGKB (2 total citations)	1 (0.5%)	1 (1%)	1 (1%)	1 (0.5%)
American College of Medical Genetics and Genomics (ACMG; 10 total citations)	10 (5%)	0 (0%)	9 (6%)	1 (0.5%)
International Statements or Guidelines (1 total citation)	0 (0%)	1 (1%)	1 (1%)	0 (0%)
Peer-reviewed literature (207 total citations)	132 (61%)	75 (57%)	78 (53%)	129 (65%)
Industrial or market solution ^a^ (13 total citations)	2 (1%)	11 (8%)	9 (6%)	4 (2%)
Clinical Pharmacogenetics Implementation Consortium (CPIC; 12 total citations)	6 (3%)	6 (5%)	9 (6%)	3 (2%)
News article (1 total citation)	0 (0%)	1 (1%)	1 (1%)	0 (0%)
Canadian Agency for Drugs and Technologies in Health (CADTH; 2 total citations)	1 (0.5%)	1 (1%)	1 (1%)	1 (0.5%)
International Council for Harmonisation of Technical Requirements for Pharmaceuticals for Human Use (ICH; 1 total citation)	0 (0%)	1 (1%)	1 (1%)	0 (0%)
International Society of Psychiatric Genetics (1 total citation)	0 (0%)	1 (1%)	1 (1%)	0 (0%)
Agency for Healthcare Research and Quality (AHRQ; 1 total citation)	1 (0.5%)	0 (0%)	0 (0%)	1 (0.5%)
Payer Technology Evaluation Center (4 total citations)	4 (2%)	0 (0%)	0 (0%)	4 (2%)
PGx test provider webpage (3 total citations)	3 (1%)	0 (0%)	0 (0%)	3 (2%)
Academic resources ^b^ (1 total citation)	1 (0.5%)	0 (0%)	0 (0%)	1 (0.5%)
Association for Molecular Pathology and/or College of American Pathologists (6 total citations)	4 (2%)	2 (2%)	2 (1%)	4 (2%)
ClinKey (1 total citation)	1 (0.5%)	0 (0%)	0 (0%)	1 (0.5%)
Emergency Care Research Institute (ECRI) Institute (7 total citations)	7 (3%)	0 (0%)	0 (0%)	7 (4%)
Hayes Knowledge Center (6 total citations)	6 (3%)	0 (0%)	0 (0%)	6 (3%)
Payer health guidelines (4 total citations)	3 (1%)	1 (1%)	2 (1%)	2 (1%)
Subject Matter Panel and Advisory Committee ^c^ (4 total citations)	2 (1%)	2 (2%)	3 (2%)	1 (0.5%)
American Association for Clinical Chemistry (1 total citation)	0 (0%)	1 (1%)	0 (0%)	1 (0.5%)
American Psychiatric Association (1 total citation)	0 (0%)	1 (1%)	0 (0%)	1 (0.5%)
International Society of Psychiatric Genetics (1 total citation)	0 (0%)	1 (1%)	0 (0%)	1 (0.5%)
Government agency health technology assessment (2 total citations)	0 (0%)	2 (2%)	0 (0%)	2 (1%)
National Society of Genetic Counselors (1 total citation)	1 (0.5%)	0 (0%)	1 (1%)	0 (0%)

^a^ Company advertising and selling operational or consultative services and solutions to healthcare service providers. ^b^ Academic-derived teaching or reference tool for healthcare providers and researchers. ^c^ Convened panel or committee of subject matter experts.

**Table 2:** Key findings or conclusions within psychiatry-specific RWE studies (*n* = 6) cited more than once and in more than one payer policy and at the top Oxford Centre for Evidence-Based Medicine 2011 (OCEBM) Evidence Levels observed across all policies.

Study PMID (OCEBM Evidence Level)	Publication Year	Payer Coverage Decision(s)	Key Findings or Conclusions
22198443 (Level 3)	2012	Not Covered (2 policies)	Poor metabolizers and ultra-rapid metabolizers received significantly higher chlorpromazine equivalent doses than extensive metabolizers and intermediate metabolizers. There was a tendency that the increase primarily was caused by CYP2D6-dependent antipsychotics and not as expected by CYP2D6-independent antipsychotics.
23047243 (Level 3)	2012	Both Covered (1 policy) and Not Covered (3 policies)	A greater reduction in overall Quick Inventory of Depressive Symptomatology, Clinician Rated (QIDS-C16), and Hamilton Rating Scale for Depression (HAM-D17) scores were achieved with PGx-guided treatment.
24018772 (Level 3)	2013	Both Covered (1 policy) and Not Covered (1 policy)	Study replicated the magnitude of effect previously observed in a prior smaller prospective pilot study (23047243). Reduction in depression scores from the baseline to the 8-week visit was greater in the PGx-guided group than in the PGx-unguided group.
25686762 (Level 1)	2015	Both Covered (1 policy) and Not Covered (1 policy)	8-week improvement in depressive symptoms in the three studies assessed displayed the same trend, with clinical outcomes differing overall as a function of the most severely categorized medication patients was prescribed at the study baseline.
26445691 (Level 3)	2015	Both Covered (1 policy) and Not Covered (1 policy)	Majority of patients showed clinically measurable improvement (rated as very much improved, much improved, or minimally improved), with most demonstrating clinically significant improvement. Among individuals with ≥ 2 prior treatment failures, the majority showed clinically measurable improvement. Patients also reported significant decreases in depression, anxiety, and medication side effects and increases in quality of life.
29690793 (Level 1)	2018	Both Covered (1 policy) and Not Covered (2 policies)	At present, there are insufficient data to support the widespread use of combinatorial pharmacogenetic testing in clinical practice, although there are clinical situations in which the technology may be informative, particularly in predicting side effects.

PMID: PubMed identification number (https://pubmed.ncbi.nlm.nih.gov/ (accessed on 25 May 2025)).

## Data Availability

The original contributions presented in this study are included in the article/Supplementary Material. Further inquiries can be directed to the corresponding authors.

## Supplementary Materials

The following supporting information can be downloaded at: https://www.mdpi.com/article/10.3390/jpm15060232/s1, Supplementary File S1: Table of Pharmacogenomic Biomarkers in Drug Labeling and Corresponding Clinical Pharmacogenetics Implementation Consortium Information, and Supplementary File S2: Oxford Centre for Evidence-Based Medicine 2011 Levels of Evidence [8].
