# Supplementary material for: An Assessment of Real-World Evidence and Other Sources Supporting Payer Coverage Decisions for Pharmacogenomic Testing in Psychiatry
Source: J Pers Med. 2025 Jun 3;15(6):232. doi: 10.3390/jpm15060232 (PMC12194790; doi:10.3390/jpm15060232)
Supplement: Supplementary file 1 [file jpm-15-00232-s001.zip › Supplementary File S1.pdf]

Table of Pharmacogenomic Biomarkers in Drug Labeling and Corresponding Clinical Pharmacogenetics Implementation Consortium Information								
Table of Pharmacogenomic Biomarkers in Drug Labeling - Psychiatry | FDA (Downloaded on 12/18/2024 from https://www.fda.gov/drugs/science-and-research-drugs/table-pharmacogenomic-biomarkers-drug-labeling)			Clinical Pharmacogenetics Implementation Consortium (CPIC; Downloaded on 12/18/2024 from https://cpicpgx.org/genes-drugs/)					
**Drug**	**Biomarker†**	**Labeling Sections**	**CPIC Guideline**	**CPIC Level**	**CPIC Level Status**	**PharmGKB Level of Evidence**	**PGx on FDA Label**	**CPIC Publications (PMID)**
Citalopram	CYP2C19	Dosage and Administration, Warnings, Clinical Pharmacology	https://cpicpgx.org/guidelines/cpic-guideline-for-ssri-and-snri-antidepressants/	A	Final	1A	Actionable PGx	25974703; 37032427
Escitalopram	CYP2C19	Adverse Reactions	https://cpicpgx.org/guidelines/cpic-guideline-for-ssri-and-snri-antidepressants/	A	Final	1A	Actionable PGx	25974703; 37032427
Amitriptyline	CYP2D6	Precautions	https://cpicpgx.org/guidelines/guideline-for-tricyclic-antidepressants-and-cyp2d6-and-cyp2c19/	A	Final	1A	Actionable PGx	23486447; 27997040
Atomoxetine	CYP2D6	Dosage and Administration, Warnings and Precautions, Adverse Reactions, Drug Interactions, Use in Specific Populations, Clinical Pharmacology	https://cpicpgx.org/guidelines/cpic-guideline-for-atomoxetine-based-on-cyp2d6-genotype/	A	Final	1A	Actionable PGx	30801677

Table of Pharmacogenomic Biomarkers in Drug Labeling and<br>Corresponding Clinical Pharmacogenetics Implementation Consortium Information								
Table of Pharmacogenomic Biomarkers in Drug Labeling - Psychiatry | FDA (Downloaded on 12/18/2024 from https://www.fda.gov/drugs/science-and-research-drugs/table-pharmacogenomic-biomarkers-drug-labeling)			Clinical Pharmacogenetics Implementation Consortium (CPIC; Downloaded on 12/18/2024 from https://cpicpgx.org/genes-drugs/)					
Nortriptyline	CYP2D6	Precautions	https://cpicpgx.org/guidelines/guideline-for-tricyclic-antidepressants-and-cyp2d6-and-cyp2c19/	A	Final	1A	Actionable PGx	23486447; 27997040
Paroxetine	CYP2D6	Drug Interactions, Clinical Pharmacology	https://cpicpgx.org/guidelines/cpic-guideline-for-ssri-and-snri-antidepressants/	A	Final	1A		25974703; 37032427
Vortioxetine	CYP2D6	Dosage and Administration, Clinical Pharmacology	https://cpicpgx.org/guidelines/cpic-guideline-for-ssri-and-snri-antidepressants/	A	Final	1A	Actionable PGx	37032427
