# Supplementary material for: An Assessment of Real-World Evidence and Other Sources Supporting Payer Coverage Decisions for Pharmacogenomic Testing in Psychiatry
Source: J Pers Med. 2025 Jun 3;15(6):232. doi: 10.3390/jpm15060232 (PMC12194790; doi:10.3390/jpm15060232)
Supplement: Supplementary file 1 [file jpm-15-00232-s001.zip › Supplementary File S2.pdf]

Oxford Centre for Evidence-Based Medicine 2011 Levels of Evidence

Question	Step 1 (Level 1*)	Step 2 (Level 2*)	Step 3 (Level 3*)	Step 4 (Level 4*)	Step 5 (Level 5)
**How common is the problem?**	Local and current random sample surveys (or censuses)	Systematic review of surveys that allow matching to local circumstances**	Local non-random sample**	Case-series**	n/a
**Is this diagnostic or monitoring test accurate?** (Diagnosis)	Systematic review of cross sectional studies with consistently applied reference standard and blinding	Individual cross sectional studies with consistently applied reference standard and blinding	Non-consecutive studies, or studies without consistently applied reference standards**	Case-control studies, or "poor or non-independent reference standard**	Mechanism-based reasoning
**What will happen if we do not add a therapy?** (Prognosis)	Systematic review of inception cohort studies	Inception cohort studies	Cohort study or control arm of randomized trial*	Case-series or case-control studies, or poor quality prognostic cohort study**	n/a
**Does this intervention help?** (Treatment Benefits)	Systematic review of randomized trials or *n*-of-1 trials	Randomized trial or observational study with dramatic effect	Non-randomized controlled cohort/follow-up study**	Case-series, case-control studies, or historically controlled studies**	Mechanism-based reasoning
**What are the COMMON harms?** (Treatment Harms)	Systematic review of randomized trials, systematic review of nested case-control studies, *n*-of-1 trial with the patient you are raising the question about, or observational study with dramatic effect	Individual randomized trial or (exceptionally) observational study with dramatic effect	Non-randomized controlled cohort/follow-up study (post-marketing surveillance) provided there are sufficient numbers to rule out a common harm. (For long-term harms the duration of follow-up must be sufficient.)**	Case-series, case-control, or historically controlled studies**	Mechanism-based reasoning
**What are the RARE harms?** (Treatment Harms)	Systematic review of randomized trials or *n*-of-1 trial	Randomized trial or (exceptionally) observational study with dramatic effect			
**Is this (early detection) test worthwhile?** (Screening)	Systematic review of randomized trials	Randomized trial	Non -randomized controlled cohort/follow-up study**	Case-series, case-control, or historically controlled studies**	Mechanism-based reasoning

*** Level may be graded down on the basis of study quality, imprecision, indirectness (study PICO does not match questions PICO), because of inconsistency between studies, or because the absolute effect size is very small; Level may be graded up if there is a large or very large effect size.**

**** As always, a systematic review is generally better than an individual study.**

How to cite the Levels of Evidence Table

OCEBM Levels of Evidence Working Group*. "The Oxford 2011 Levels of Evidence".

Oxford Centre for Evidence-Based Medicine. http://www.cebm.net/index.aspx?o=5653

** OCEBM Table of Evidence Working Group = Jeremy Howick, Iain Chalmers (James Lind Library), Paul Glasziou, Trish Greenhalgh, Carl Heneghan, Alessandro Liberati, Ivan Moschetti, Bob Phillips, Hazel Thornton, Olive Goddard and Mary Hodgkinson*
